# TakoTsubo Syndrome: First an Acute Coronary Vasculitis and Then Prolonged Myocarditis?

**DOI:** 10.31083/j.rcm2305152

**Published:** 2022-04-26

**Authors:** Olivia C Girolamo, Sven Y Surikow, Gao-Jing Ong, Thanh Ha Nguyen, Angela M Kucia, Yuliy Y Chirkov, John D Horowitz

**Affiliations:** ^1^Basil Hetzel Institute for Translational Research, University of Adelaide, 5011 Adelaide, Australia; ^2^Northern Adelaide Local Health Network, Adelaide, Australia; ^3^Central Adelaide Local Health Network, Adelaide, Australia; ^4^University of South Australia, Adelaide, Australia

**Keywords:** TakoTsubo Syndrome, vasculitis, myocarditis, inflammation

## Abstract

Since its initial description by Japanese investigators 30 years ago, TakoTsubo Syndrome (TTS) has variously been regarded as a form of acute coronary syndrome and also as a form of cardiomyopathy (or more accurately, a myocarditis). There is actually good evidence that TTS embodies both of these concepts, and the main purpose of this review is to present data that they occur sequentially. The initial phase of the disorder (over perhaps the first 48 hours post onset of symptoms) represents a form of vasculitis, with associated damage to the endothelial glycocalyx and associated permeabilization of blood vessels. This is followed by a more prolonged phase of myocardial inflammation and oedema, associated with inflammatory activation and energetic impairment within the entire myocardium. Although this phase subsides after several months, it may be followed by longstanding impairment of myocardial function, reflecting residual fibrosis. Understanding of this gradual transition in TTS pathogenesis from vasculature towards myocardium remains an important limitation of patient management, especially as many patients are still told that their hearts have “recovered” within 1–2 weeks. A number of important uncertainties remain. These include development of specific early and ongoing therapeutic strategies to be used to match the sequential pathogenesis of TTS.

“And so these men of Indostan

Disputed loud and long,

Each in his own opinion

Exceeding stiff and strong,

Though each was partly in the right,

And all were in the wrong!”

From: Six wise men of Hindustan

## 1. Introduction: What’s in a Name?

John Godfrey Saxe’s poem expresses the dilemma experienced by individuals who attempt to reconstruct an entity from a fragment. Just as the six blind men in the traditional Indian story each failed to grasp the overall nature of the elephant by feeling one of its parts, so the overall nature of TakoTsubo Syndrome has posed a considerable challenge to our understanding, one that persists to this day.

TakoTsubo Syndrome (TTS; TakoTsubo Cardiomyopathy, Apical Ballooning Syndrome, Broken Heart Syndrome) was first described in full clinical detail by Japanese investigators in 1990 [1]. Typically, the left ventricle during the acute phase of TTS exhibits apical hypokinesis with some degree of basal hyperkinesis, thus resembling a Japanese octopus trap (“takotsubo”).

From the beginning, TTS may have been assumed to be a form of acute coronary syndrome (ACS) because it usually presented with prolonged chest pain, was often associated with infarct-like electrocardiographic (ECG) changes, engendered release of markers of cardiomyocyte injury, and in many cases led to the early development of severe hypotension with something resembling cardiogenic shock [2].

On the other hand, some major differences from “conventional” ACS were present from the start. Most patients were women, usually of advanced years, and mostly with few other conventional coronary risk factors [3, 4, 5]. Emergency coronary angiography usually showed no hemodynamically significant coronary stenoses, or at most, coronary disease which was anatomically non-congruent with the areas of hypokinesis within the left ventricle [6, 7]. Furthermore, serial echocardiography suggested rapid improvement of left ventricular function, suggesting that TTS might be a transient condition [8].

Therefore, when the time came (as it did immediately!) to ascribe cause to this phenomenon, there was great uncertainty, which persists today. Essentially, there are different pathogenetic views which may be summarised as follows:

(1) TTS is an acute coronary syndrome, with regional heterogeneity of myocardial impact.

(2) TTS is a myocardial inflammatory disorder, with no appreciable “supply-side” pathology.

The purpose of this review is to present the data in favour of these two extreme seemingly irreconcilable views, and then to discuss possible “compromise” pathogenetic constructs for TTS. One of the potential benefits of such a “hybrid view” of pathogenesis is that it might help explain heterogeneity, both of onset and of recovery.

## 2. TTS As an Acute Coronary Syndrome: An Ongoing Debate Now Related to the Initial Clinical Phase of TTS

Perhaps the concept that TTS is an ACS arises from the usual mode of clinical presentation of the disorder (with chest pain mimicking an evolving myocardial infarction (MI)). The Japanese investigators who initially described TTS, noting absence of fixed coronary artery disease, eventually suggested that the pathogenesis might be multivessel coronary artery spasm (CAS) [1]. In support of this concept, multivessel CAS has been described in a number of reports, both in Japanese and Caucasians [3, 9, 10, 11, 12, 13, 14, 15], while conversely among patients with documented CAS there have also been a number of cases of myocardial necrosis [16].

On the other hand, in most cases CAS is a symptomatically fluctuating disorder with random episodes of prolonged chest pain, which only occasionally lead to appreciable myocardial cell death [17]. Furthermore, the distribution of left ventricular hypokinesis/akinesis in the most common form of TTS (apical hypokinesis) is non-concordant with that of the coronary arteries which supply the apex [18]. Furthermore, a substantial proportion of TTS patients have hypokinesis within the apices of their right ventricles, again a finding which is not congruent with any anatomical postulate [19]. Similarly, it would be difficult to suggest anatomical bases for the less frequent mid-ventricular or inverted patterns of TTS [18].

How then has the “ACS theory” survived the last 30 years? Conventionally, the precipitation of ACS has been ascribed to rupture/fissure of atheromatous plaques, with consequent platelet aggregation and thrombus formation [20]. Indeed patients with CAS often have crises related to platelet-rich thrombus formation without ruptured plaques (“plaque fissure”) [21], but evidence of such a process in the majority of TTS patients is lacking. As regards the occurrence of CAS in association with TTS, evidence falls into two categories: incidental observations and specific evaluations of coronary physiology. There have been sporadic case reports of CAS in association with TTS [3, 10, 22, 23, 24, 25, 26]. Studies seeking inducibility of CAS are summarised in Table 1 (Ref. [3, 11, 12, 13, 14, 15, 25]).

**Table 1.** **Previous reports of pharmacologically induced CAS in association with TTS diagnosis**.

Author/year	Phase of test	Inductor	Patients tested (n)	% CAS	Site(s)/Focal: diffuse (n)
Tsuchihashi *et al*., 2001 [15]	Subacute	ACh	48	21%	RCA (n = 2)
					LCA (n = 3)
					RCA+LCA (n = 5)
Kurisu *et al*., 2002 [3]	Acute (n = 6)	ACh (n = 12)	14	Acute: 67%	Epicardial (n = 4)
	Subacute (n = 8)	Erg (n = 2)		Subacute: 75%	Multivessel (n = 6)
Athanasiadis *et al*., 2006 [11]	Subacute	ACh	17	59%	3:7
Yoshida *et al*., 2007 [12]	Acute	ACh	6	50%	Epicardial (n = 2)
					Multivessel (1)
Misumi *et al*., 2010 [25]	Acute	Erg	1	100%	RCA
Uchida *et al*., 2010 [13]	Acute	ACh	8	75%	LAD (n = 3)
					D1 branch (n = 2)
					D2 branch (n = 1)
Verna *et al*., 2018 [14]	Acute	ACh	47	85%	13:34

ACh, acetylcholine; Erg, ergonovine; LCA, left coronary artery; RCA, right coronary artery; LAD, left anterior descending; D1, first diagonal branch of LDA; D2, second diagonal branch of LAD.

From the Table 1 data, there is evidence that some patients exhibit inducible CAS during the acute phase of TTS. Several of the studies documenting induction of CAS in a substantial proportion of patients in the acute stage of TTS come from Japanese groups. This is of relevance given there is substantial evidence that East Asian populations are more prone to the development of CAS, especially in male smokers within this population [27]. However, another factor predisposing this East Asian propensity towards CAS may be loss-of-function mutations of mitochondrial aldehyde dehydrogenase (${\mathrm{ALDH}}_{2}$) which are present in approximately 30% of East Asians [28] and which may diminish cell defences to oxidative stress [29]. It is also possible that a larger proportion of patients develop problems with coronary blood flow either because of acute impairment of left ventricular relaxation (and therefore extramural compression of coronary arteries during diastole), because of disturbed coronary rheology, and/or because of acute impairment of coronary vasodilator reserve [30]. De Caterina *et al*. [31] have also demonstrated that in the acute stages of TTS, there is not only a reduction in rates of contrast flow down the left anterior descending coronary artery, but also that this impairment exceeds the anomaly seen post MI. These data strongly suggest an early phase of increased microvascular resistance, although it is not clear to what extent such an increase reflects anatomic changes as distinct from increases in vasoconstrictor tone.

As regards impaired rheology, we have previously demonstrated a substantial increase in plasma concentrations of the glycocalyx component syndecan-1 (SD-1) during the first few days after onset of symptoms of TTS [32]. SD-1 levels had returned to normal within 3 months [32]. Zhang *et al*. [33] have demonstrated that integrity of the vascular glycocalyx is important for maintenance of the Starling equilibrium of fluid status between intravascular and extravascular distribution. This combination of increased vascular permeability and fluid extravasation would theoretically contribute to impaired coronary perfusion. Additionally, loss of glycocalyx would induce non-laminar coronary flow, with an implicit increase in coronary vascular resistance, and a stimulus towards increased vascular expression of thioredoxin interacting protein (TXNIP), which acts as a competitive inhibitor of nitric oxide (NO) effect and an activator of the NLRP3 inflammasome [34]. Interestingly, the early phase of TTS is also associated with supranormal tissue responsiveness to NO as assessed via inhibition of platelet aggregation [35]. Increased NO response may contribute to incremental vascular permeability and development of severe hypotension/shock in the first 24 hours post onset of symptoms.

Therefore, a **consensus view** would be that in the acute stages of TTS there is impairment of coronary flow rates, reduction of coronary vasodilator reserve (as assessed via adenosine injection) [31] and in a substantial proportion of patients, inducible CAS. However, it is not known whether these changes are secondary to acute disruption of the endothelial glycocalyx, or whether they primarily represent altered vasomotor reactivity.

It remains important to ascertain what contribution (if any) ischaemia makes to the acute haemodynamic state in TTS. For example, it is now known that TTS is associated with impaired myocardial energetics [36], and it is theoretically possible that coronary vasoconstriction, or spasm, might contribute to this energetic disturbance. However, all energetic studies so far reported have been performed sub-acutely [36, 37] when it is far less likely that spasm would persist. It is feasible but unproved that incremental sympathetic discharge and catecholamine release, documented in the early stages of TTS [38], might tend to provoke ischemia via induction of microvascular constriction and increases in myocardial work. Indeed, hypertension has occasionally been reported in the early stages of TTS [39]. However, surges in catecholamine release contribute both to redox stress and to glycocalyx “shedding”, with associated fluid and leukocyte extravasation [40]. Thus, catecholamines can theoretically contribute to microvascular dysfunction, intravascular volume depletion and hypotension, and to the establishment of extracellular oedema in the heart, as well as pleural and pericardial cavities.

## 3. TTS as Myocarditis/Cardiomyopathy

The majority of publications before 2015 refer to TTS as a “cardiomyopathy”. This nomenclature theoretically abrogates any vascular component of pathogenesis, including myocardial ischaemia consequent on regional or global coronary dysfunction. To the best of our knowledge, no specific reason has ever been advanced to support the “cardiomyopathy” designation. However, there is no doubt that TTS is characterised by potentially reversible bi-ventricular dysfunction without a clear-cut ischaemic precipitant. Table 2 summarises the available arguments in favour of a primary coronary vasomotor versus a distinct myopathic pathogenesis for TTS.

**Table 2.** **Summary of categoric arguments regarding the pathogenesis of TTS**.

A. Arguments in favour of “ACS”		
	(1) Clinical presentation with chest pain, ECG changes and myocardial necrosis.	
	(2) Acutely disordered coronary haemodynamics	
		(a) Retardation of coronary blood flow rate,
		(b) Variable inducibility of CAS
	(3) Damage to endothelial glycocalyx	
B. Arguments in favour of myocarditis/cardiomyopathy		
	(1) Regionally impaired ventricular systolic function with transient reduction in LVEF and prolonged reduction in GLS	
	(2) Impaired myocardial energetics	
	(3) Inflammatory changes within the myocardium:	
		(a) Macrophage infiltration
		(b) Increased TXNIP expression → ${\mathrm{NLRP}}_{3}$ inflammasome activation
		(c) Nitrosative stress within the myocardium
	(4) Eventual (variable) myocardial fibrosis	

ACS, acute coronary syndrome; CAS, coronary artery spasm; GLS, global longitudinal strain; TXNIP, thioredoxin interacting protein.

The concept of TTS as a “cardiomyopathy” owes much to the work of Holger Nef and colleagues. For example, in 2007 the group reported results of myocardial biopsy (taken from the intraventricular septum) in the acute stage of 8 TTS patients [41]. These showed: (1) substantial myocardial accumulation of glycogen, (2) cell swelling of myocardial cells and damaged mitochondria, (3) cell debris in interstitial spaces, (4) accumulation of macrophages and (5) proliferation of fibroblasts. It was speculated that this represented primarily the result of local effects of catecholamine secretion, but ischaemic damage was not excluded.

Most interestingly, Nef and colleagues became fascinated with the minimal extent of myocardial necrosis in most patients with TTS. In 2009, they published a biochemical analysis of mechanisms whereby myocardium might be protected against extensive necrosis. Both acute and sub-acute biopsies were performed on 16 patients, revealing activation of the PI3K/AKT signalling pathway (RISK pathway), which is also activated by NO [42]. This activation was transient and was not seen in sub-acute cases. These findings emphasise the possibility that contractile disturbances in TTS might result primarily from transient metabolic/energetic impairment during the acute phase of the condition. On the other hand, these studies did not, at any stage, focus on extent of oxidative stress within the myocardium during the acute phase, potential activation of reactive oxygen species mediating such stress, or the implications of myocardial infiltration with both macrophages and neutrophils, despite noting its presence. There was also no investigation of myocardial expression of humoral mediators of inflammatory activation.

Surikow *et al*. [43] reported post-mortem cardiac findings in patients dying during the acute stages of TTS. Results were notable for increased tissue expression of TXNIP and formation of 3-nitrotyrosine (3-NT), a “fingerprint” of peroxynitrite (${\mathrm{ONOO}}^{-}$)-induced tyrosine nitration [44], and thus of the inflammatory and DNA-damaging effects of ${\mathrm{ONOO}}^{-}$. Similar findings were subsequently obtained from detailed examination of the myocardium in a rat model of TTS, in which macrophage infiltration of the myocardium was documented together with increased myocardial content of TXNIP and of 3-NT [45].

The capacity of in situ imaging of the heart with cardiac MRI (CMR) can be enhanced by specific modalities designed to quantitate interstitial fluid overload (such as T2-weighted images and T-1 mapping [46]) or adapted to detect fibrosis. It should be emphasised that the early performance of CMR (say 3 days post onset of TTS) is increasingly recognised as the best means of ensuring that TTS is not misdiagnosed as “Type 2 AMI”. Furthermore, addition of a magnetic resonance spectroscopy (MRS) package can be utilised to measure myocardial concentrations of ATP and phosphocreatine (PCr) [47]. The PCr:ATP ratio represents extent of availability of PCr as a transportable source of cardiac energetics [47]. A number of studies have shown that after acute attacks of TTS, there is prolonged excessive T2-weighted signal from the myocardium, indicating fluid overload [48]. This could be taken as evidence of inflammatory oedema, but as outlined above it might also be a transudate due to vascular permeation. Importantly, however, increased myocardial T2-signal persists for at least 3 months after attacks [36, 48]. Results of simultaneous phosphorus MRS studies in TTS patients were first reported by Dawson and co-workers, and have shown that there is impairment of myocardial cellular energetics for at least 4 months post-acute attacks [36]. This raises the issue of the implications of persistent impairment of quality of life after onset of TTS [49], and points to a current deficiency in the literature related to evaluation of exercise performance in the long-term post attacks. Recently, Scally and colleagues have demonstrated the development of variable degrees of myocardial fibrosis in the long-term of TTS consistent with prolonged activation of pro-fibrotic mechanisms such as the transient expression of myofibroblasts [38].They have also reported the results of CMR studies performed together with injection of ultrasmall superparamagnetic particles of iron oxide [50], which confirm that there is extensive macrophage-mediated infiltration of myocardium post TTS attacks.

All studies performed to investigate pathophysiology of the human myocardium during TTS are limited by difficulties of tissue access, and therefore a variety of animal and tissue models of TTS have been developed. The earliest approach was taken by Ueyama and colleagues, who utilised immobilisation stress in rats to induce both ECG and echocardiographic changes similar to those seen in TTS [51]. They reported beneficial effects from autonomic blockage and, in ovariectomised female rats, from oestrogen supplementation. A variety of other rodent models (both rat and mice) of TTS have now been developed, all of them utilising “pulse” exposure to high concentrations of catecholamines. The catecholamine most frequently used is the non-specific β-adrenoceptor agonist isoprenaline (isoproterenol: ISO). Indeed Paur *et al*. [52] reported in a landmark study in 2012 that induction of TTS-like contractile abnormalities in rats following catecholamine exposure was associated with the development of β_2_-adrenoceptor stimulation biased towards Gi-based post-receptor signalling, which could be abolished by the selective antagonist pertussis toxin.

Using exogenous infusion of catecholamines in a rat model, Shao *et al*. [53] confirmed the role of Gi-protein signalling in TTS in inducing negative inotropy and subsequent cardiac contractile dysfunction, whilst simultaneously contributing a cardioprotective effect in counteracting effects of β-adrenoreceptor-Gs overstimulation. These findings place TTS particularly in juxtaposition with “catecholamine cardiomyopathy” [54], without fully exploring the asymmetry of left ventricular dysfunction.

Gi-stimulation is also linked to activation of nitric oxide synthase (NOS) [55]. This brings to question the pathophysiological impact of nitrosative stress in the presence of increased NOS activation and subsequent NO production in combination with superoxide ($O_{2}$^–^) formation to generate ${\mathrm{ONOO}}^{-}$. Surikow *et al*. [45] sought to evaluate the role of nitrosative stress, quantitated via tyrosine nitration in acute TTS using a rat model induced by ISO. The findings confirmed the development of nitrosative stress (by the presence of 3-NT), and of PARP-1 activation as a mediator of impaired myocardial energetics [45].

The concept that occurrence of a myocarditis is an inherent early component of the pathogenesis of TTS remains problematic to this day. For many years, myocarditis was listed as an exclusion criterion for the diagnosis of TTS [56], and only very recently have the various guidelines specifically referred to viral myocarditis, rather than myocarditis in general, as an exclusion criterion [57, 58]. However, a substantial grey area remains here, and has recently been brought into sharp relief by reports of association between COVID infection and “TTS”, essentially centred upon the finding of myocarditis with associated chest pain [59]. It therefore seems likely that the increased utilization of CMR imaging as a component of the diagnostic algorithm for TTS will resolve some issues, and especially differentiation from AMI, while focusing on others, such as other causes of myocarditis.

## 4. TTS as a Form of Initial and Transient ACS, Transmuting into a More Prolonged Myocarditis/Cardiomyopathy

Whilst TTS may initially appear to mimic a conventional ACS, the underlying pathogenetic mechanisms are complex in nature, pertaining to components of both vascular endothelial damage and ongoing myocardial inflammation. And so, it appears we are faced with a conundrum similar to that of The Blind Men and The Elephant. Currently, each interpretation of the condition’s pathogenesis is taken in isolation of the opposing views, thus seeming diametrically opposite and irreconcilable. In light of this, we propose the need for hybrid pathogenetic construct which integrates the view of TTS occurring initially as a transient ACS, and eventually transmuting into a prolonged myocarditis.

**Fig. 1.**
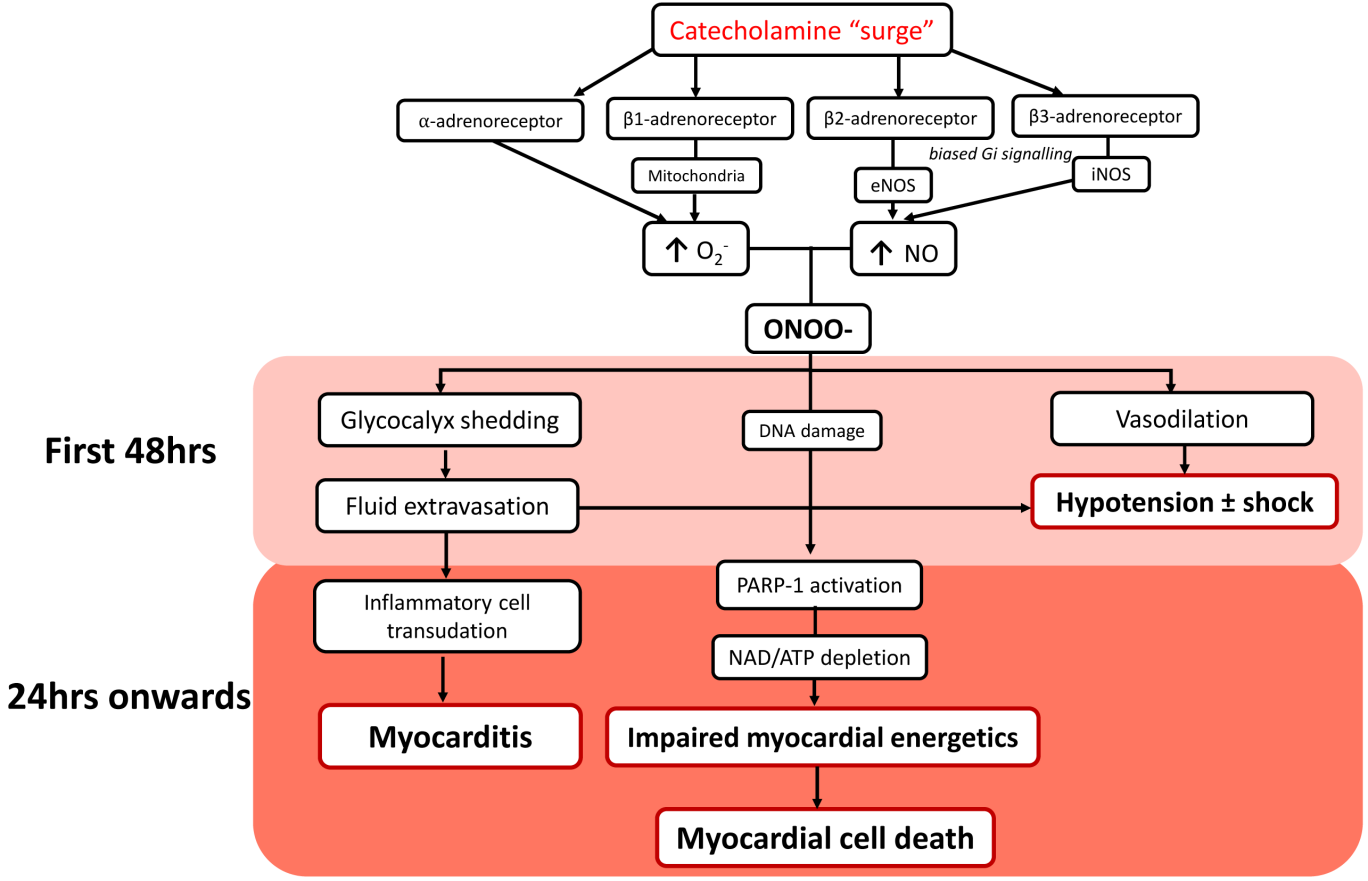
**Schematic depiction of the sequence of vascular dysfunction followed by prolonged myocarditis, as key pathogenetic components in TTS**. $O_{2}$^–^, Superoxide anion; NO, nitric oxide; ${\mathrm{ONOO}}^{-}$, peroxynitrite anion; PARP-1, Poly (ADP/ribose polymerase-1). Pink = early events, predominantly in vasculature; red = later events, predominantly in myocardium. Note that coronary constriction may well occur within the first 24 hours, despite systemic vasodilatation, and that this may reflect the impact of glycocalyx shedding.

The only consensus regarding the pathogenesis of TTS is the involvement of catecholamine release as a stimulus. Our proposed pathogenic construct, schematically depicted in Fig. 1, is founded by this catecholamine “surge”, which can occur either exogenously [60] or endogenously; clinically seen as an acute stress response [2, 58]. Catecholamines act primarily to induce a paradoxical cardioprotective-impairment of inotropic status via overstimulation of the α- and β-adrenoreceptors within the cardiomyocyte [60, 61], and to stimulate increased $O_{2}$^–^ production, inducing oxidative stress [62]. Additionally, the intrinsic linkage of β2- and β3-adrenoreceptors to NOS results in its activation, and subsequent increase in NO production [63], which in the presence of increased $O_{2}$^–^, spontaneously forms ${\mathrm{ONOO}}^{-}$, acting as a mediator of nitrosative stress [64]. We believe this to be a crucial point in the biochemical cascade, with the concurrent effects of both oxidative and nitrosative stress resulting in the myocardial dysfunction characterising TTS, largely reflecting glycocalyx shedding [32], oedema and myocardial inflammation [48], and impaired cardiac energetics [36, 37].

Disruption to the endothelial glycocalyx layer has been previously linked to ${\mathrm{ONOO}}^{-}$ formation [65] and may theoretically contribute to both myocardial oedema associated with TTS (via fluid extravasation) and inflammatory cell transudation and subsequent myocarditis. However, perhaps the most important implication of ${\mathrm{ONOO}}^{-}$ in TTS is its role in inducing DNA damage and triggering the activation of the nuclear enzyme poly(ADP-ribose) polymerase (PARP-1) [66]. In the case of TTS, it is proposed that acute nitrosative stress results in extreme DNA and the hyperactivation of PARP-1, causing significant loss of NAD+ and reductions in cellular ATP, establishing an “energy sink” which may directly impair cardiac energetics [67].

Fig. 1 is a schematic representation of key aspects of TTS pathophysiology, as currently recognised, with emphasis on the potential for a sequential process, initially affecting vasculature and then myocardium.

## 5. How Then Shall We Educate Patients and Their Carers?

There is great need that clinicians taking care of patients recovering from TTS have a good understanding of the cause of the disorder (including the lack of association with coronary atherogenesis), the frequently slow and incomplete recovery from symptoms of residual lassitude and exertional dyspnoea, the risk of recurrence and the currently limited understanding of therapeutics for this disorder.

It remains frequent for patients to be discharged from hospital after approximately 3 days, and to be told that “everything has returned to normal”. When these patients (as frequently the case) have slowly resolving symptoms, they become anxious that something has gone wrong with their recoveries [68].

Although there are some data favouring long-term utility of treatment with ACE inhibitors and angiotensin-receptor blockers, largely as regards reduction in risk of recurrence [69, 70], many patients are not prescribed these, and instead receive aspirin, statins or β-adrenoceptor antagonists, for which there is little or no supporting evidence.

Patients often become particularly anxious about prevention of recurrences, and avoid activities which they perceive as likely to engender recurrences [71]: these may involve physical activities and/or operations. However, it is far from sure to which extent the recurrence of TTS is predictable, and therefore clinician advice is limited at present in this regard.

Finally, many patients become depressed after attacks of TTS, and are appropriately treated for this complication. However, it is important to recognise that some antidepressants may precipitate attacks of TTS, given that their mechanisms of action result in increases in catecholamine concentrations in the synaptic cleft between sympathetic nerve-endings and myocardial β-adrenoceptors [72]. Therefore prescription of antidepressants requires appropriate knowledge by treating clinicians.

In conclusion, rather than representing either an acute coronary syndrome or a cardiomyopathy alone, TTS remains a complex, incompletely understood condition [73] revolving around initial vascular followed by myocardial inflammatory changes, and often resulting in prolonged patient debility. Given that it is far from rare, greater understanding will facilitate appropriate advice and treatment for affected patients.
